# Admission glucose is a significant outcome predictor in anterior circulation stroke: approaching the sweet spot

**DOI:** 10.1186/s42466-025-00393-0

**Published:** 2025-06-10

**Authors:** Alexandra Filipov, Martin Andermann, Guilherme Lepski, Analía Arévalo, Tim Hilgenfeld, Silvia Schönenberger, Christoph Gumbinger, Markus Möhlenbruch, Peter Arthur Ringleb, Jessica Jesser

**Affiliations:** 1 Department of Neurology, Heidelberg University Hospital, Heidelberg, Germany; 2 Department of Experimental Surgery, Medical School, University of São Paulo, São Paulo, Brazil; 3 Department of Neuroradiology, Heidelberg University Hospital, Im Neuenheimer Feld 400, 69120 Heidelberg, Germany

**Keywords:** Admission glucose, Hyperglycemia, Mechanical thrombectomy

## Abstract

**Background:**

Admission glycemia has emerged as an important outcome predictor in the context of mechanical thrombectomy (MT) for large vessel occlusions (LVO) in ischemic stroke. However, a clinically relevant threshold of glucose levels to identify patients at risk for poor functional outcome has yet to be established.

**Methods:**

We conducted a retrospective, monocentric, consecutive registry-based analysis of patients who underwent MT for anterior circulation LVO. Good outcome was defined as functional independence after 90 days (90d mRS < 3) or no deterioration from premorbid mRS. We performed a multiple logistic regression analysis to assess the association between admission glucose levels and functional outcome, including for well-established outcome predictors, i.e. age, NIHSS, Alberta Stroke Program Early CT Score (ASPECTS), time to reperfusion, unsuccessful recanalization, presence of bleeding, and diabetes. In addition, we conducted a receiver operating characteristic (ROC) analysis to determine the optimal admission glucose threshold that best discriminates patients at risk for poor outcome, maximizing sensitivity and specificity.

**Results:**

We analyzed 509 patients (mean age = 74.3 ± 12.6 years, median previous mRS = 1.5, 48% male). 194 patients (38.1%) had good outcome and 315 (61.9%) had poor outcome. According to the logistic regression admission glucose (*p* = 0.012, OR 1.009 95% CI [1.002 1.016]) contributed to predicting poor outcome, while known diabetes did not show a significant contribution. The ROC analysis revealed an admission glucose of 117 mg/dL (59.7% sensitivity; 58% specificity) to be the optimal cut-off value to discriminate patients at risk for poor outcome with an OR of 2.3.

**Conclusion:**

Admission hyperglycemia is an independent predictor of poor outcome after MT for LVO in the anterior circulation. We hypothesize, that optimal glucose values in patients undergoing MT will likely be in the low normoglycemic range. Prospective controlled studies with targeted glucose values will be needed for validation.

## Introduction

Currently, mechanical thrombectomy (MT) is considered standard of care in acute ischemic stroke for patients with large vessel occlusions (LVO) in the anterior circulation [1]. Sudden reperfusion of large ischemic brain tissue areas, however, is associated with known risks: in ischemic cells, increased glucose delivery can induce oxidative stress, which can induce cell death and lead to the phenomenon known as reperfusion damage after successful MT [2]. Admission glycemia has emerged as a significant outcome predictor in patients undergoing MT for LVO in ischemic stroke. Several studies have investigated the relationship between admission serum glucose levels and various clinical outcomes after MT, establishing a correlation between high glucose levels and poor functional outcome [3, 4] as well as high mortality and bleeding complications [5]. An analysis of the HERMES Collaboration data revealed that MT treatment effects were more favorable for patients with glucose levels below 100 mg/dL [6]. Nevertheless, a definitive threshold for admission glycemia levels associated with favorable versus unfavorable outcomes has yet to be established.

The aims of our study were (1) to investigate the influence of admission glucose on functional outcome after LVO stroke and MT, and (2) to determine a clinically relevant cut-off value for glucose levels that would help us identify patients at risk for poor outcome.

## Methods

We conducted a retrospective analysis on ischemic stroke patients with large vessel occlusion in the anterior circulation treated with mechanical thrombectomy. Data were collected from a single-center prospective consecutive registry with 90 days follow up. The local institutional ethics review committee approved the collection and analysis of anonymized registry data.

Patients with acute ischemic stroke and large vessel occlusion in the anterior circulation who underwent at least one thrombectomy attempt between 01/2015 and 02/2020 were chosen for study inclusion. Patients who had missing data on one or more of the variables reported below were excluded from statistical analyses. Good outcome was defined as functional independence at 90 days (90d mRS 0–2) or no deterioration from previous mRS and poor outcome was defined as functional dependency at 90 days and deterioration from the premorbid functional level, or death.

### Statistical analyses

Between-group differences were assessed using *t* tests for metric variables and Mann–Whitney *U* tests for ordinal and categorical variables as appropriate. We then performed a multiple logistic regression analysis, including the following established predictors: age (years), sex (male/female), previous mRS (0–4), diabetes (yes/no), arterial hypertension (yes/no), coronary heart disease (yes/no), atrial fibrillation (yes/no), hypercholesterolemia (yes/no), time to admission (min), NIHSS score at admission, glucose level at admission (mg/dL), thrombolysis (yes/no), ASPECTS (0—10), time from puncture to reperfusion (min), poor TICI (< 2b), number of thrombectomies, and bleeding complication on a follow up CT scan (yes/no). The number of patients per predictor exceeded the recommended minimum number of 10–20 [7, 8]. No outliers had to be removed from the data (criterion: > 3 SD; [9]); all predictors followed linear relationships, as revealed with the Box-Tidwell procedure ( [10] all Bonferroni-corrected *p*’s > 0.025), and low intercorrelations among the predictors indicated that multicollinearity was not a confounding issue among our predictors (all *r*’s <<|0.800|). Goodness-of-fit was checked using the Hosmer-Lemeshow test, revealing a model fit with sufficient quality for interpretation (*χ*² (8) = 3.631, *p* = 0.889).

Finally, receiver operating characteristic (ROC) analyses were conducted using the *fitglm* and *perfcurve* functions in MATLAB^®^ R2022b (The MathWorks, Inc., Natick, MA, USA); the corresponding confidence intervals (CI_BCa_) were bias-corrected and accelerated as suggested by Efron & Tibshirani (1993), based on 5,000 resamples [11].

Statistical analyses were conducted using SPSS^®^ 27.0 (IBM, Armonk, New York, NY, USA).

## Results

### Baseline characteristics

We found 751 patients with acute ischemic stroke and large vessel occlusion in the anterior circulation who underwent at least one thrombectomy attempt between 01/2015 and 02/2020. 242 patients with missing data on one or more of the variables were excluded. A total of 509 patients were included in the statistical analyses.

**Fig. 1:**
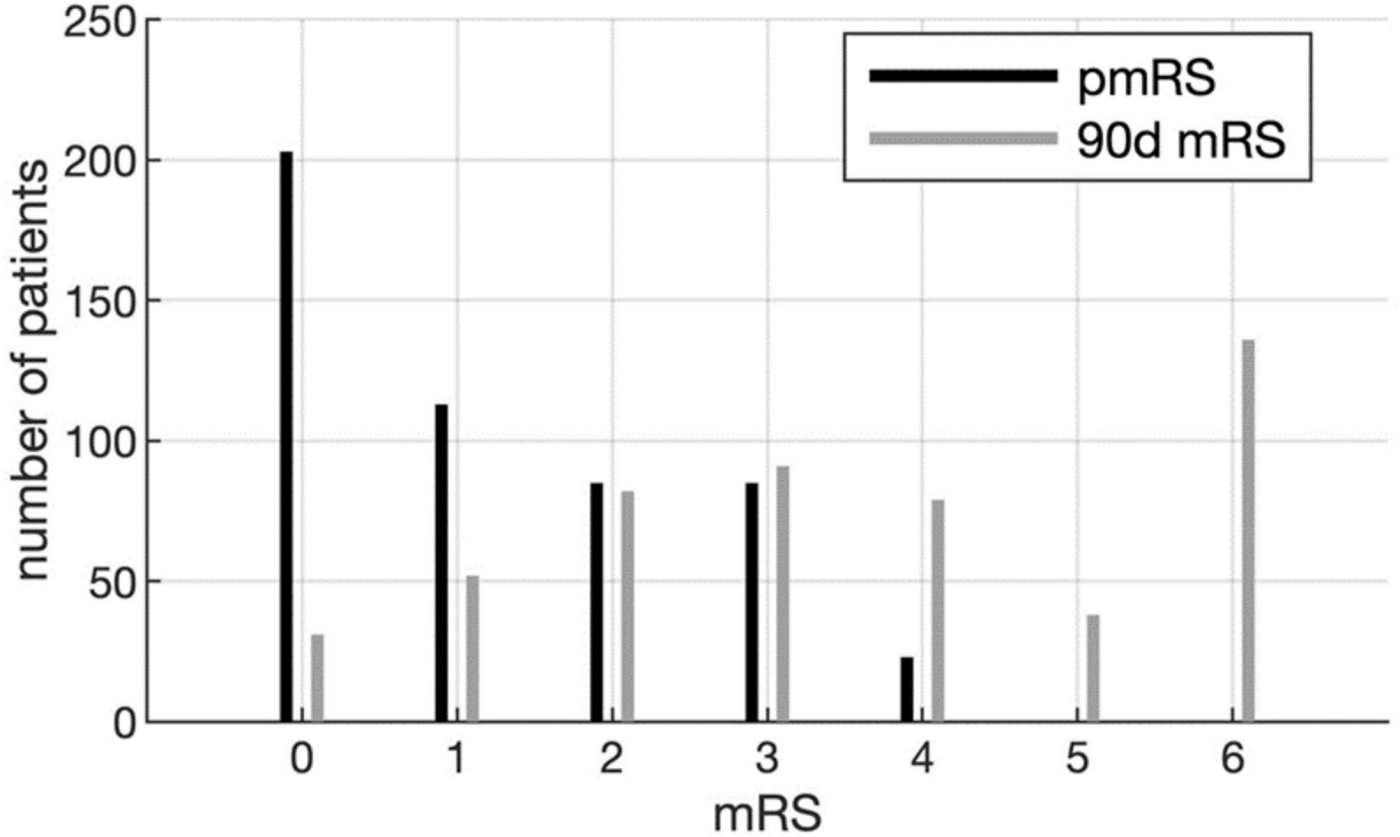
Shift in functional outcome at 90 days. pmRS and 90d mRS scores are denoted with black and grey lines, respectively

In our cohort of 509 patients (mean age = 74.3 ± 12.6 years, median previous mRS = 1.5, 21.2% premorbid functional dependency, 47.9% male), 194 patients (38.1%) had good outcome, and 315 (61.9%) had poor outcome. The mRS distribution at 90 days is presented in Fig. 1.

Patients with poor outcome were older (mean age in years (SD) 76.4 (11.8) vs. 70.9 (13.2), *p* < 0.001), had a more pronounced premorbid impairment (previous mRS median (IQR min) 0 (2) vs. 1 (2) *p* < 0.001), and suffered more often from diabetes (27.9% vs. 20.1%, *p* = 0.048). Groups did not differ significantly from each other in terms of any other comorbidities or premedication. Time to admission was longer in patients with poor outcome (median (IQR min), 197 (279.75) vs. 168.5 min (198), *p* = 0.039). Also, patients with poor outcome had worse neurological deficits at admission, based on the NIHSS (median (IQR min) 20 (9) vs. 13 (12), *p* < 0.001) and more extensive early signs of infarction on an initial CT scan (ASPECTS, median (IQR min) 9 (3) vs. 9 (2), *p* < 0.001).

**Fig. 2:**
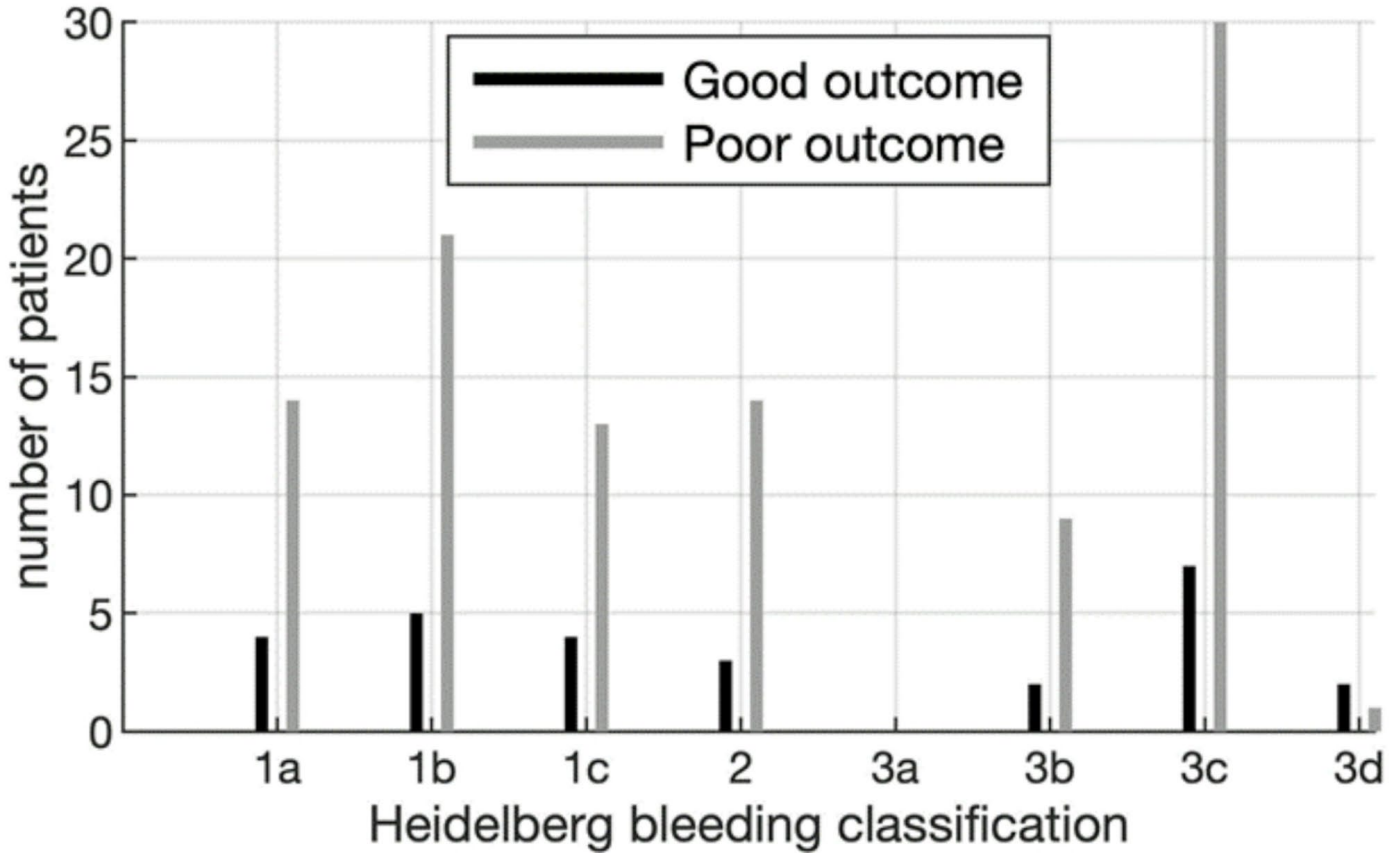
Distribution of intracranial hemorrhage according to Heidelberg classification in patients with good vs. poor outcome

Evidence of bleeding on a follow-up CT scan was more often found in patients with poor outcome (38.1% vs. 17.0%, *p* < 0.001). Subarachnoid hemorrhage was the most prevalent subtype of intracranial bleeding after reperfusion therapy, according to the Heidelberg classification [12], and was associated with poor outcome (see Fig. 2).

Poor outcome was more frequent in patients with unsuccessful recanalization indicated by TICI < 2b (n (%) 56 (17.8) vs. 8 (4.1), *p* < 0.001), in patients with more thrombectomy maneuvers (median (IQR) 2 (2) vs. 1 (1)) and if time to reperfusion was longer (median (IQR) [min] 76 (77.5) vs. 48 (43), *p* < 0.001).

**Fig. 3:**
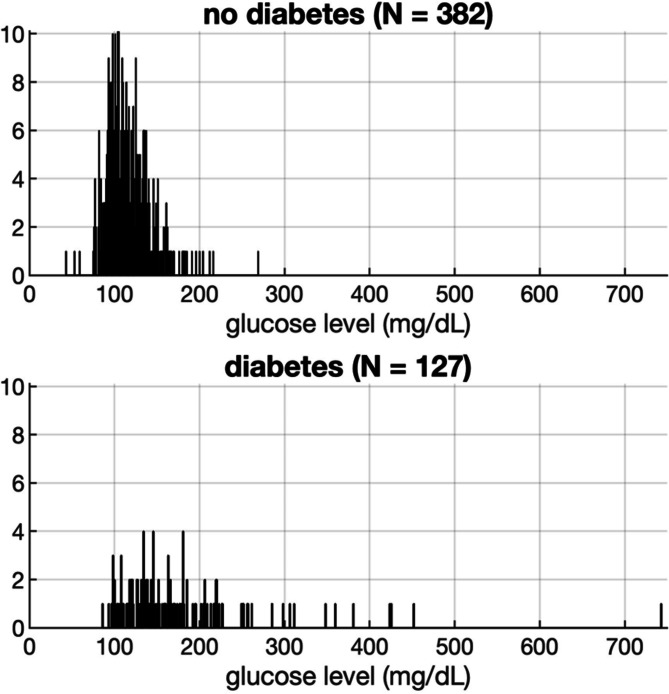
Distribution of admission glucose values amongst patients with and without known diabetes

Finally, higher admission glucose levels were found in patients with poor outcome (mean (SD) [mg/dL] 138.9 (63.8) vs. 119.6 (35.1), *p* < 0.001). Baseline characteristics are presented in Table 1. In our cohort, 127 patients (25%) had a pre-existing diagnosis of diabetes. Patients with known diabetes had higher admission glucose values than patients without diabetes (t (507) = 12.213, *p* < 0.001). Admission glucose levels in patients with and without known diabetes are presented in Fig. 3.

**Table 1:** Baseline characteristics

Parameter	All patients	Good outcome (90d mRS < 3 or no deterioration)	Poor outcome (90d mRS ≥ 3)	*p*
	**509**	**194 (38.1)**	**315 (61.9)**	
Age, mean (SD) [years]	74.3 (12.6)	70.9 (13.2)	76.4 (11.8)	< 0.001***
Male, *n* (%)	244 (47.9)	102 (52.6)	142 (45.1)	0.100
pmRS, median (IQR)	1 (2)	0 (2)	1 (2)	< 0.001***
Diabetes, *n* (%)	127 (25.0)	39 (20.1)	88 (27.9)	0.048*
Hypertension, *n* (%)	399 (78.4)	151 (77.8)	248 (78.7)	0.812
Coronary heart disease, *n* (%)	136 (26.7)	45 (23.2)	91 (28.9)	0.159
Atrial fibrillation, n (%)	247 (48.5)	87 (44.9)	160 (50.8)	0.193
Hypercholesterolemia, *n* (%)	170 (33.4)	65 (33.5)	105 (33.3)	0.968
Antithrombotic agent, *n* (%)	112 (22.0)	41 (21.1)	71 (22.5)	0.710
Oral anticoagulation, *n* (%)	106 (20.8)	40 (20.6)	66 (21.0)	0.928
Time to admission, median (IQR) [min]	188 (248.75)	168.5 (198)	197.0 (279.75)	0.039*
NIHSS score, median (IQR)	17 (11)	13 (12)	20 (9)	< 0.001***
NIHSS score, mean (SD)	16.4 (7.7)	13.3 (7.4)	18.3 (7.3)	< 0.001***
Glucose, mean (SD) [mg/dL]	131.5 (55.4)	119.6 (35.1)	138.9 (63.8)	< 0.001***
Thrombolysis, *n* (%)	269 (52.9)	108 (55.7)	161 (51.1)	0.317
ASPECTS, median (IQR)	9 (3)	9 (2)	9 (3)	< 0.001***
ASPECTS, mean (SD)	8.5 (1.5)	8.9 (1.3)	8.2 (1.6)	< 0.001***
Time to reperfusion, median (IQR) [min]	61 (68.25)	48 (43)	76 (77.5)	< 0.001***
TICI < 2b, *n* (%)	64 (12.6)	8 (4.1)	56 (17.8)	< 0.001***
Number of thrombectomies, median (IQR)	1 (2)	1 (1)	2 (2)	< 0.001***
Bleeding, *n* (%)	153 (30.0)	33 (17.0)	120 (38.1)	< 0.001***
90d mRS, median (IQR)	3 (4)	2 (1)	5 (2)	< 0.001***

Baseline characteristics. pmRS, premorbid Rankin Scale; mRS, modified Rankin Scale; d, day; NIHSS, National Institutes of Health Stroke Scale; ASPECTS, Alberta Stroke Program Early CT Score; TICI, thrombolysis in cerebral ischemia; *n*, number of patients; %, percentage of quality; SD, standard deviation; IQR, interquartile range; min, minutes. Significant *p*-values are denoted in bold; *, *p* < 0.05; ***, *p* < 0.001

### Logistic regression

The binomial logistic regression model was highly significant, *χ*²(17) = 182.790, *p* < 0.001***, resulting in a moderate amount of explained variance that corresponds to a medium sized effect, as indicated by Nagelkerke’s *R*² = 0.410 [13]. Overall classification accuracy was 75.6%, with sensitivity = 0.838 and specificity = 0.624. Statistical model information is presented in Table 2. Higher age, higher NIHSS scores, higher glucose levels, lower ASPECTS, longer time to reperfusion, unsuccessful recanalization (TICI < 2b) and the presence of bleeding strongly contributed to predicting poor outcome after stroke. Notably, time to admission only shortly missed significance as a predictor; sex, previous mRS, hypertension, coronary heart disease, atrial fibrillation, hypercholesterolemia, thrombolysis, number of thrombectomy maneuvers, and diabetes did not show significant contributions.

**Table 2:** Logistic regression model

Predictor	regression coefficient	standard error	Wald	*p*	odds ratio [95% CI]
Age	0.048	0.011	17.937	< 0.001***	1.049 [1.026 1.072]
Male sex	0.091	0.238	0.148	0.701	1.096 [0.687 1.747]
pmRS	0.158	0.102	2.408	0.121	1.171 [0.959 1.429]
Diabetes	0.003	0.310	0.000	0.993	1.003 [0.546 1.841]
Hypertension	-0.167	0.314	0.285	0.593	0.846 [0.457 1.564]
Coronary heart disease	0.278	0.269	1.069	0.301	1.320 [0.780 2.236]
Atrial fibrillation	-0.317	0.247	1.650	0.199	0.729 [0.449 1.181]
Hypercholesterolemia	-0.028	0.250	0.013	0.910	0.972 [0.596 1.586]
Time to admission	0.001	0.000	2.948	0.086	1.001 [1.000 1.002]
NIHSS score	0.086	0.017	25.373	< 0.001***	1.090 [1.054 1.128]
Glucose	0.009	0.003	6.261	0.012*	1.009 [1.002 1.016]
Thrombolysis	-0.010	0.250	0.002	0.968	0.990 [0.607 1.615]
ASPECTS	-0.249	0.088	7.982	0.005**	0.780 [0.656 0.927]
Time to reperfusion	0.007	0.003	4.865	0.027*	1.007 [1.001 1.013]
TICI < 2b	1.708	0.475	12.945	< 0.001***	5.519 [2.176 13.996]
Number of thrombectomies	0.180	0.098	3.366	0.067	1.197 [0.988 1.451]
Bleeding	0.890	0.268	11.003	0.001**	2.436 [1.439 4.122]

Logistic regression model information. df = 1 for all Wald statistics. pmRS, premorbid Rankin Scale; NIHSS, National Institutes of Health Stroke Scale; ASPECTS, Alberta Stroke Program Early CT Score. Significant *p*-values are denoted in bold; *, *p* < 0.05; **, *p* < 0.01; ***, *p* < 0.001

### ROC analysis

**Fig. 4:**
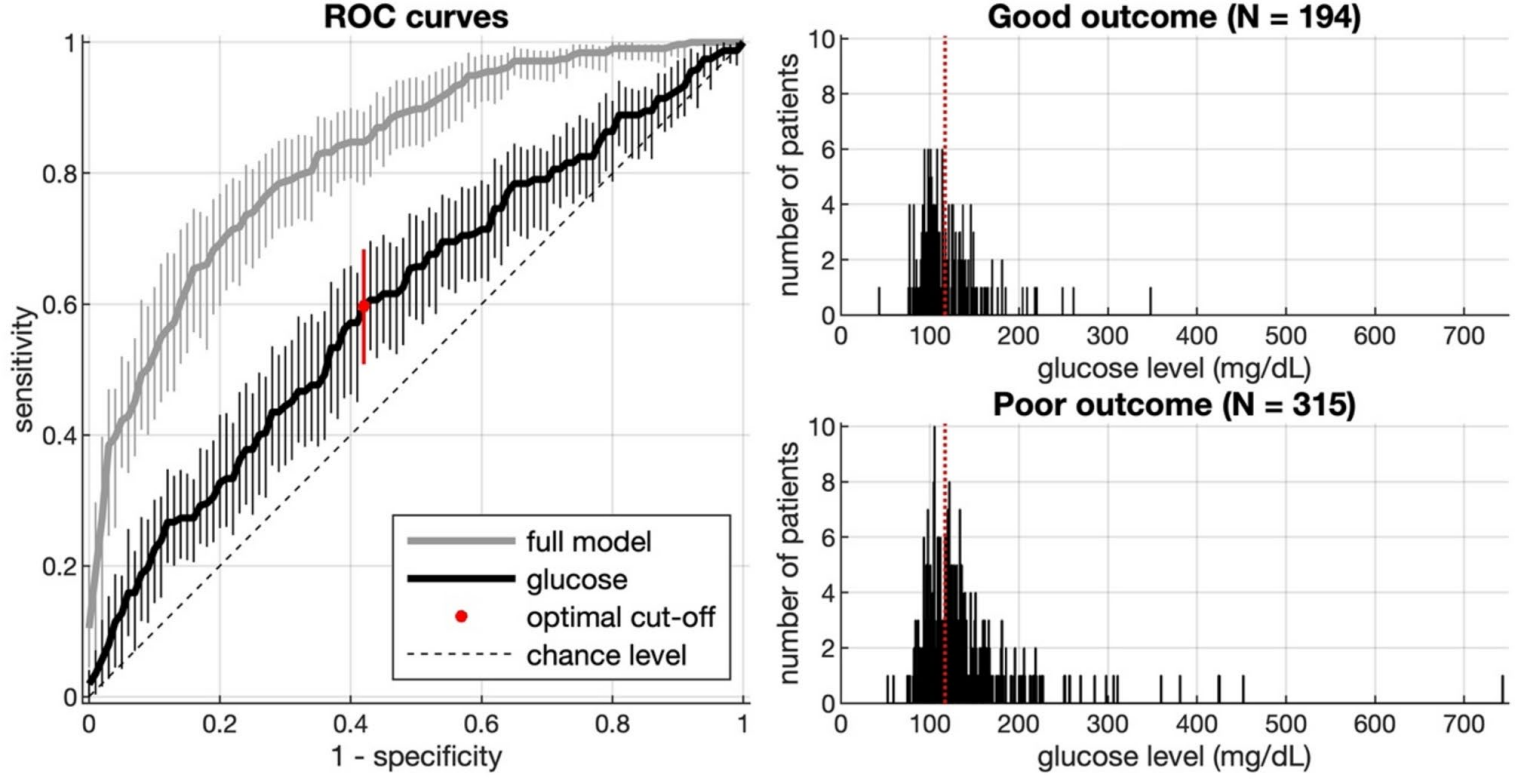
The left panel presents ROC curves for the full logistic regression model with all predictors (grey) and for a model with glucose level as the only predictor (black); error bars denote 95% CI_BCa_ (Efron & Tibshirani, 1993), based on 5,000 resamples. Regarding the latter model, the optimal cut-off is indicated with a red circle; in the right panels of the figure, the dotted red lines illustrate how this relates to the distribution of glucose levels among patients with good vs. poor outcome

The grey line in the left panel of Fig. 4 presents the ROC curve for the full logistic regression model with all predictors; this model yielded an area under the curve (AUC) of 0.830 (95% CI_BCa_: [0.790 0.862]). In the same panel, the black line denotes the ROC curve for a model with glucose level as the only predictor; here, AUC was 0.610 (95% CI_BCa_: [0.561 0.661]). The red circle indicates the point with minimum distance to perfection (i.e., [0,1]) regarding that model; using this optimal cut-off, patients with poor outcome were identified with a sensitivity of 0.597 (95% CI_BCa_: [0.509 0.687]) and a specificity of 0.580. The optimal cut-off corresponded to a glucose level of 117 mg/dL. The right panels of Fig. 4 illustrate how this cut-off (dotted red line) relates to the distribution of glucose levels among patients with good vs. poor outcome. A glucose level above 117 mg/dL had a positive predictive value of 70% for poor outcome. The negative predictive value -- meaning the probability for good outcome with an admission glucose below 117 mg/dL -- was 47%. The relative risk for poor outcome with an admission glucose above 117 mg/dL was 1.321; this translates into an OR of 2.321 for poor outcome at an admission glucose above 117 mg/dL.

## Discussion

Our data clearly confirmed that higher admission glucose levels are an independent predictor of poor functional outcome after MT for LVO in the anterior circulation. The proportion of 38.1% of stroke patients reaching functional independence at 90 days in our cohort is in line with comparable real world data [14, 15]. Interestingly, admission glycemia along with well-known powerful predictors like age, NIHSS, pre-treatment ASPECTS, poor TICI, and bleeding complications still added significance to our outcome prediction model, while a premorbid deficit, time to admission, intravenous thrombolysis and (interestingly) diabetes, did not [16, 17]. Diabetes is a well-established predictor of poor recovery after stroke [18]. These findings suggest a particularly strong impact of admission glucose on functional outcome in our patient cohort. Notably, patients with known diabetes had higher admission glucose values than patients without diabetes. Regarding the distribution of glucose levels amongst patients with diabetes (see Fig. 3), we noticed a wide range of up to 450 mg/dL, reflecting the potential of acute metabolic derailment. Specifically, patients with pre-existing diabetes are prone to stress hyperglycemia [19] in the context of a severe stroke due to catecholamine release and exacerbated insulin resistance. Nevertheless, as Fig. 3 illustrates, stress hyperglycemia can occur regardless of a pre-existing diagnosis of diabetes and is known to be associated with worse stroke outcomes [20]. Stress hyperglycemia has been found to be a predictor of poor functional recovery, mortality and intracranial hemorrhage specifically after MT for LVO [21, 22, 23]. Potential mechanisms of how acute hyperglycemia worsens the effects of cerebral ischemia, mainly deriving from animal models, have been discussed. They involve more severe cytotoxic injury after stroke, leading to greater neuronal damage, disruption of the blood-brain barrier, which can lead to brain edema and hemorrhagic transformation of infarcts, an increase in reactive oxygen species (ROS) exacerbating cellular damage by mitochondrial dysfunction, lactate accumulation and acidosis promoting cell death, elevated extracellular glutamate levels mediating excitotoxicity, and increased neuroinflammation [24]. It can be assumed that specifically in the context of MT for LVO, reperfusion injury, which is mediated by very similar mechanisms [25], leads to potentiated neuronal damage in acute hyperglycemia. Conversely, hyperglycemia does not seem to have detrimental effects in lacunar stroke [26], where reperfusion of a large ischemic brain area does not occur. However, acute hyperglycemia may lead to an underestimation of the infarct core size on CT perfusion imaging misguiding treatment decisions and promoting hemorrhagic complications after reperfusion [27]. One may thus conclude that admission glucose levels before MT for LVO should follow the rule “*the lower*,* the better*”. Meanwhile, hypoglycemia is also a driver of neuronal damage in acute ischemia due to the lack of energy supply and various associated metabolic changes, as an adrenergic response, followed by vasoconstriction and pro-coagulation [24]. This may explain the finding in clinical studies that intensive glucose lowering strategies with a risk of hypoglycemia did not show beneficial effects on stroke outcome [28].

Given the fine line between hypo- and hyperglycemic damage in the context of MT for LVO, a cut-off value that can discriminate patients at risk for poor functional recovery would be clinically relevant. Fuentes et al. (2009) found capillary blood glucose of 155 mg/dL to be independently associated with poor functional recovery after three months (53% sensitivity; 73% specificity) [29]. Notably, this was a heterogenous cohort regarding stroke type, time of admission and acute therapy. Furthermore, MT was not an established treatment at that time so that reperfusion of large ischemic brain areas was not as common in that cohort. In our more homogenous cohort of patients with anterior circulation LVO treated with MT, we found a serum admission glucose of 117 mg/dL (59.7% sensitivity; 58% specificity) to be the optimal cut-off value identifying patients at risk for poor outcome.

An admission glucose level of 117 mg/dL or higher was associated with a 2.3-fold risk of poor outcome at 90 days compared with patients with lower admission glucose.

Our results must be interpreted in light of some limitations. This was a monocentric, retrospective study based on prospectively collected registry data. Missing information was frequent but thanks to the abundance of data in the registry, we were able to analyze a solid cohort. The 90-day follow-up was not blinded for medical history. Regarding the validity of our prediction model, admission glucose levels alone predicted functional outcome with only limited sensitivity and specificity. Accordingly, our proposed cut-off value cannot be taken as irrevocable.

Nevertheless, confirming that higher admission glucose levels are an independent predictor of poor functional recovery after MT for LVO, we hypothesize– based on our findings - that optimal glucose levels for patients undergoing MT might be lower than for acute stroke patients who do not. The risk of reperfusion injury may increase with rising glycemic levels. We suggest enhanced monitoring of glucose levels in the acute phase of LVO, particularly when endovascular treatment is performed. Analyzing data from more frequent glucose monitoring in these patients could provide a better understanding of glucose’s influence on outcomes and the dynamics of glucose fluctuations in the acute stroke setting. Controlled studies comparing outcomes of patients with targeted glucose levels in the context of MT should then follow to validate our hypothesis. Considering the lack of convincing results of insulin treatment in acute stroke due to acute hypoglycemia, alternative interventions like GLP-1 receptor agonists could be an option. Along these lines, research will have to further elucidate the complex mechanisms linking hyperglycemia and stroke outcome, ultimately guiding future clinical practices.

## Data Availability

The datasets generated and/or analyzed during the current study are not publicly available due to local regulations and restrictions but are available from the corresponding author upon reasonable request.
